# Wound-Healing and Antibacterial Activities of *Caryopteris mongolica* Bunge Root Extract Ointment in an Experimental Animal Model

**DOI:** 10.4014/jmb.2605.05002

**Published:** 2026-07-09

**Authors:** Tuyajargal Iimaa, Tsenguun Tuyajargal, Namuunaa Ganbayar, Undarmaa Otgonbaatar, Stipan Nurbyek, Undrakhbayar Tserendorj, Soyolmaa Gurdorj, Altanchimeg Adilbish, Byambajav Tseesuren, Batkhuu Javzan, Davaapurev Bekh-Ochir

**Affiliations:** 1 Department of Chemical and Biological Engineering, School of Engineering and Technology, National University of Mongolia, Ulaanbaatar 14201, Mongolia; 2 Department of Medical Chemistry, School of Bio Medicine, Mongolian National University of Medical Sciences, S. Zorig Street, Ulaanbaatar 14210, Mongolia; 3 Institution of Biomedical Sciences, Mongolian National University of Medical Sciences, S.Zorig Street, Ulaanbaatar 14210, Mongolia; 4 Graduate School of Medical Science, Mongolian National University of Medical Sciences, S.Zorig Street, Ulaanbaatar 14210, Mongolia; 5 Institute of Veterinary Medicine, Mongolian University of Life Sciences, Ulaanbaatar 17042, Mongolia

**Keywords:** *Caryopteris mongolica* Bunge, Bioactive compounds, Ointment formulation, Histopathology, Tissue regeneration, Antibacterial activity

## Abstract

*Caryopteris mongolica* Bunge is a medicinal plant native to Mongolia with a long history of use in traditional medicine for treating skin infections and inflammatory conditions. Recent phytochemical investigations have identified potent antibacterial constituents in its root extracts, particularly active against Gram-positive bacteria. The present study aimed to develop a novel topical ointment formulation incorporating ethanolic extract of *C. mongolica* roots, Vaseline, and lavender oil, and to evaluate both its antibacterial activity and wound-healing efficacy within an integrated experimental framework. Antibacterial activity was assessed using disk diffusion method against four Gram-positive strains (*Staphylococcus aureus*, *Staphylococcus epidermidis*, *Enterococcus faecalis*, and *Micrococcus luteus*). Activity was first observed at 3.0 mg/mL, and consistent inhibition at 5.0 mg/mL, which was selected as the working concentration. Physicochemical evaluation showed that all ointment parameters were within USP 30 standards, and microbiological testing indicated the absence of pathogenic contaminants. Wound-healing efficacy was evaluated using eight-week-old BALB/c mice randomly divided into three groups (n = 15 per group): untreated control, vehicle ointment control, and *C. mongolica* ointment-treated group. Topical application every two days for 14 days resulted in higher wound closure in the *C. mongolica*-treated group (97.1%) compared with vehicle (92.96%) and untreated (92.35%) controls. Statistically significant differences were observed during the early phase of healing (days 3 and 5; *p* < 0.05), whereas no significant differences were detected at later time points. Histopathological analyses showed improved epidermal regeneration (794.65 ± 75.27 μm vs. 394.59 ± 58.5 μm), granulation tissue thickness, collagen deposition, and neovascularization in treated wounds, along with minimal inflammatory cell infiltration by day 14. These findings suggest that *C. mongolica* root extract ointment is associated with antibacterial activity and improved wound-healing outcomes in an experimental animal model, though a direct causal relationship between these two properties was not established.

## Introduction

Traditional Mongolian medicine, with a documented history of more than 5,000 years, represents one of the most ancient and sophisticated traditional healing systems in Asia. It has developed through centuries of accumulated knowledge on the relationship between humans, animals, and the environment. This system continues to serve as an important component of public health, providing safe, effective, and affordable healthcare for both preventive and therapeutic purposes. In recent years, traditional medicine has also become an important source for modern pharmacological research and drug discovery, particularly in the identification of bioactive natural compounds with clinically relevant biological activities [1, 2].

Medicinal plants are a central component of traditional pharmacopoeias worldwide, and their therapeutic potential has been widely recognized across different cultures. Natural products derived from plants remain important in modern drug development due to their broad pharmacological activities, including antibacterial, antioxidant, anti-inflammatory, anticancer, hepatoprotective, and analgesic effects. Compared with synthetic drugs, plant-derived preparations often exhibit multi-target activity, improved biocompatibility, and lower toxicity. In addition, their accessibility and diversity of bioactive compounds make them valuable candidates for pharmaceutical development. As a result, plant-based products are widely used in various formulations, including extracts, essential oils, capsules, tablets, and topical preparations for the treatment of infectious and chronic diseases [3-8].

Wound healing is a highly dynamic and complex biological process that has become a major focus in modern biomedical research due to its clinical and socioeconomic impact. Chronic and non-healing wounds, such as diabetic ulcers, pressure sores, and traumatic injuries, remain major global health challenges and may lead to severe complications including infection, amputation, and reduced quality of life [9-12]. Consequently, increasing attention has been directed toward natural bioactive compounds and biomaterial-based dressings capable of enhancing tissue repair, reducing inflammation, and preventing microbial infection. A number of medicinal plants, including *Aloe vera*, *Centella asiatica*, *Terminalia chebula*, *Tephrosia purpurea*, *Carica papaya*, and *Hippophae rhamnoides*, have demonstrated significant wound-healing properties. These effects are mainly attributed to phytochemicals such as flavonoids, tannins, saponins, and polysaccharides, which promote fibroblast proliferation, collagen synthesis, angiogenesis, and antioxidant defense mechanisms during tissue repair [3-8].

Mongolia, characterized by its vast steppes and unique biodiversity, harbors a rich diversity of medicinal plants. Traditional Mongolian knowledge, rooted in nomadic culture, has long recognized the therapeutic potential of native flora for treating pain, inflammation, skin infections, and respiratory diseases. Among these, *Caryopteris mongolica* Bunge (*C. mongolica*) is a valuable medicinal species native to Mongolia and northern China. It has traditionally been used to treat conditions such as pain, edema, rheumatism, arthritis, cough, sore throat, bronchitis, colds, and eczema [13-15]. The widespread use of *C. mongolica* in traditional medicine underscores its potential as a multipurpose therapeutic agent with broad biological activity. Phytochemical studies on *C. mongolica* have identified numerous secondary metabolites that contribute to its pharmacological effects. Flavonoids and essential oils extracted from this plant have demonstrated anti-inflammatory, antioxidant, and antimicrobial properties [16-18]. Root extracts, in particular, have exhibited potent antibacterial activity against pathogenic microorganisms, suggesting potential applications in topical formulations for wound disinfection and healing [19-21]. Furthermore, chemical analyses have revealed the presence of diverse bioactive compounds such as iridoid glycosides, alkaloids, and abietane-type diterpenoids, which are associated with strong antibacterial and anti-inflammatory actions, neuroprotective effects, cytotoxic activity against human leukemia HL60 cells, and inhibitory activity against *Brucella* species [22-29].

The bioactive compounds of *C. mongolica* may be associated with enhanced wound healing through multiple mechanisms, including inhibition of bacterial growth, modulation of inflammatory responses, and stimulation of fibroblast proliferation. Phytochemicals from medicinal plants are well known to regulate inflammation, promote collagen synthesis, enhance angiogenesis, and accelerate epithelialization, thereby supporting tissue regeneration [30-32]. In addition, essential oils of *C. mongolica* may further contribute to healing by improving local circulation and reducing inflammatory responses in damaged tissues. Despite these promising findings, no previous study has systematically developed a standardized topical formulation of *C. mongolica* root extract and evaluated its combined antibacterial and wound-healing effects in an *in vivo* model. Therefore, the aim of this study was to develop a topical ointment containing *C. mongolica* root extract through systematic formulation optimization and to evaluate, within a single integrated framework, both the antibacterial activity and wound-healing potential of the formulation in a murine excisional model, with particular relevance to the management of infection-complicated wounds.

## Materials and Methods

### Plant Extract Preparation

**Fig. 1:**
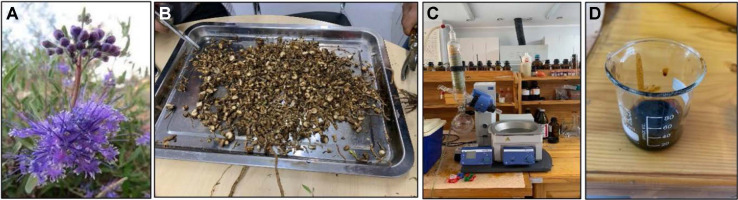
Extraction of plants. (**A**) *C. mongolica*, (**B**) dried roots of *C. mongolica*, (**C**) A rotary vacuum evaporator set at 40°C at 100 rpm, and (**D**) the root extract of *C. mongolica*.

Roots of *Caryopteris mongolica* Bunge were collected from Batsumber soum, Tuv province, Mongolia. A voucher specimen of the plant material was authenticated and deposited at the Laboratory of Bioorganic Chemistry and Pharmacognosy, School of Engineering and Technology, National University of Mongolia. Fresh roots (250 g) were washed with distilled water to remove dust, cut into 3−5 mm pieces, and air-dried at room temperature. The dried materials were extracted with 96% ethanol at a 1:10 (w/v) ratio and kept at room temperature for 3−5 days with occasional stirring. The resulting mixture was filtered, and the filtrate was evaporated at 40°C under reduced pressure using a rotary vacuum evaporator operated at 100 rpm, followed by drying (Fig. 1). Finally, 29.3 g of crude ethanolic extract was obtained from 250 g of dried roots, corresponding to an extraction yield of 11.7% (w/w).

### HPLC Analysis

To confirm the chemical identity and batch consistency of the extract used in this study, high-performance liquid chramatography (HPLC) fingerprinting was performed. The root extract was analyzed using a Dionex UltiMate 3000 HPLC (Thermo Fischer Scientific, USA). Chromatographic separation was achieved on an Acclaim C18 (5 μm, 120 Å, 4.6 × 250 mm; Thermo Fisher Scientific) with a total run time of 35 min. The mobile phase consisted of acetonitrile (A) and 0.1% trifluoroacetic acid (TFA) in water (B), using a gradient elution profile in which the A:B ratio was changed from 1:9 to 9:1. The flow rate was 1.0 mL/min, the injection volume was 5.0 μL, and UV detection was recorded at 210 nm.

### Determination of the Optimal Concentration of Plant Extract

To determine the effective concentration range, the *C. mongolica* extract was incorporated into Original Protecting Vaselline (Unilever, South Africa) at concentrations of 0.5, 0.75, 1.0, 3.0, 5.0, 10.0, and 15.0 mg/mL. Antibacterial activity was evaluated using the agar disk diffusion method against four Gram-positive bacterial strains, including *Staphylococcus aureus*, *Staphylococcus epidermidis*, *Enterococcus faecalis* and *Micrococcus luteus*. Bacterial suspensions were adjusted to approximately 10^6^ CFU/mL (0.5 McFarland standard) and evenly spread onto nutrient agar plates. Sterile paper disks were impregnated with 50 μL of each formulation and placed on the agar surface. The plates were incubated at 37°C for 24 h, after which the diameters of the inhibition zones were measured.

### Physicochemical properties and Preparation of *C. mongolica* Ointment

Based on findings from our previous studies and other reports [1, 2, 19], which demonstrated that crude *C. mongolica* root extract exhibited potent antibacterial activity against Gram-positive bacteria at a concentration of 500 μg/disc, a final concentration of 5.0 mg/mL was selected for the ointment formulation.

### *C. mongolica* Ointment

To formulate the experimental ointment, 100 mg of the root extract was dissolved in 1 mL of 99.7% ethanol (Xilong Scientific Co., Ltd., China) and stirred for 10 minutes at room temperature until a homogeneous mixture was obtained. Subsequently, 200 μL of 100% pure *Lavandula angustifolia* (Maillette) essential oil (Hortus & Apothicus, France) was added dropwise under continuous stirring. Finally, 100% pure Vaseline (Unilever Trumbull Research Services, Inc., USA) was added to a final volume of 20 mL, yielding a root extract concentration of 5.0 mg/mL. The formulation was mixed thoroughly to ensure homogeneity.

The physicochemical properties of the ointment were evaluated using the test methods specified for Vaseline in the United States Pharmacopeia (USP 30 standards).

### Control Ointment

The control ointment was prepared using the same procedure, except that the *C. mongolica* root extract was omitted. Specifically, 200 μL of lavender essential oil was dissolved in 1 mL of ethanol under vigorous magnetic stirring at room temperature. Vaseline was then added to obtain a uniform base formulation comparable to the test ointment.

### Microbiological analysis of *C. mongolica* Ointment

Five samples of the ointment (batch No. 20080505) were submitted to the National Reference Laboratory for Food Safety, Mongolia, on 10 August 2020 for microbiological evaluation. All analyses were performed in an ISO/IEC 17025-accredited laboratory. To assess the overall microbial load of the ointment, standard enumeration tests were conducted for the total aerobic bacterial count, yeast and mold counts, and Enterobacteriaceae. Serial dilutions of each sample were cultured on appropriate media, incubated under standard conditions (30−35°C for bacteria and 20−25°C for fungi), and the results were expressed as colony-forming units per gram (CFU/g). In addition, detection tests were carried out to determine the presence of potential pathogenic microorganisms, including *Staphylococcus aureus* and *Pseudomonas aeruginosa*, using selective media and confirmatory biochemical identification.

### Antibacterial Аctivity of *C. mongolica* Ointment

The antibacterial activity of the *C. mongolica* ointment was assessed using the disk diffusion method against four Gram-positive bacterial species: *S. aureus*, *S. epidermidis*, *E. faecalis*, and *Micrococcus luteus*. Nutrient agar plates were inoculated with approximately 10^6^ CFU of bacteria, and wells measuring 6−8 mm in diameter were aseptically punched using a sterile cork borer. Each well was filled with 50 μL of the ointment, and the plates were incubated at 37°C for 24 h. The inhibition zones were then measured, and kanamycin was used as a positive control.

### Wound Creation, Animal Grouping, and Treatment Protocol

All animal procedures were approved by the Institutional Animal Care and Ethics Committee of the Institute of Veterinary Medicine, Mongolian University of Life Sciences (approval no. VSBMR20/01/15, granted 14 June 2020) and were conducted in accordance with the National Guidelines on the Care and Use of Laboratory Animals.

Healthy male BALB/c mice aged eight weeks and weighing 32.5 ± 2.5 g were acclimatized under standardized environmental conditions and randomly assigned to three groups (n = 15 per group): (1) untreated control (negative control), (2) control ointment-treated (positive control), and (3) *C. mongolica* ointment-treated (experimental group).

The dorsal region of each mouse was shaved, disinfected with 70% ethanol, and anesthetized using isoflurane vapor (Pfizer Co., UK). A full-thickness excisional wound measuring 10 mm in diameter was created on the central dorsal region. The ointments were topically applied every two days for 14 days. Wound areas were photographed and analyzed using ImageJ software to calculate wound contraction rates. To monitor potential adverse effects of the ointment, changes in body weight of the experimental animals were recorded throughout the study period.

### Histological Sample Processing and Scoring

Permission number of animal etics committee of Mongolian University of Life Sciences is VSBMR 20/01/15. Procedures of euthanization: At predetermined intervals (days 1, 3, 5, 7, and 14), mice were humanely euthanized by exposure to excess isoflurane vapor (approximately 1–2 mL) until respiration ceased and death was confirmed. Skin tissue samples were then immediately collected for histological analysis. Samples were fixed in 10% neutral-buffered formalin, embedded in paraffin, and sectioned at a thickness of 4 μm. Sections were stained with hematoxylin and eosin (H&E) and examined under a light microscope (Olympus BX53JP, Japan) to assess histopathological changes related to wound healing. Collagen deposition was semi-quantitatively scored on a 0-4 scale as follows: 0, no detectable collagen fibers; 1, scant, disorganized collagen fibers; 2, moderate collagen deposition with partial organization; 3, abundant, well-organized parallel collagen bundles; and 4, dense, mature collagen architecture comparable to normal dermis. Inflammatory cell infiltration was scored using the same 0−4 scale: 0, no inflammatory cells; 1, minimal infiltration; 2, moderate infiltration; 3, marked infiltration; and 4, severe, extensive infiltration. All histological procedures were performed at the Pathology Laboratory of the Institute of Veterinary Medicine, Mongolia.

### Statistical Analysis

A *p*-value less than 0.05 was considered statistically significant. Normality of the residuals from each one-way ANOVA model was assessed using the Shapiro-Wilk test prior to interpretation. Given the limited sample size at each time point (n = 3 per group), Kruskal-Wallis tests were additionally performed as a non-parametric cross-check of the ANOVA results.

## Results

### HPLC Chromatographic Fingerprinting

**Fig. 2:**
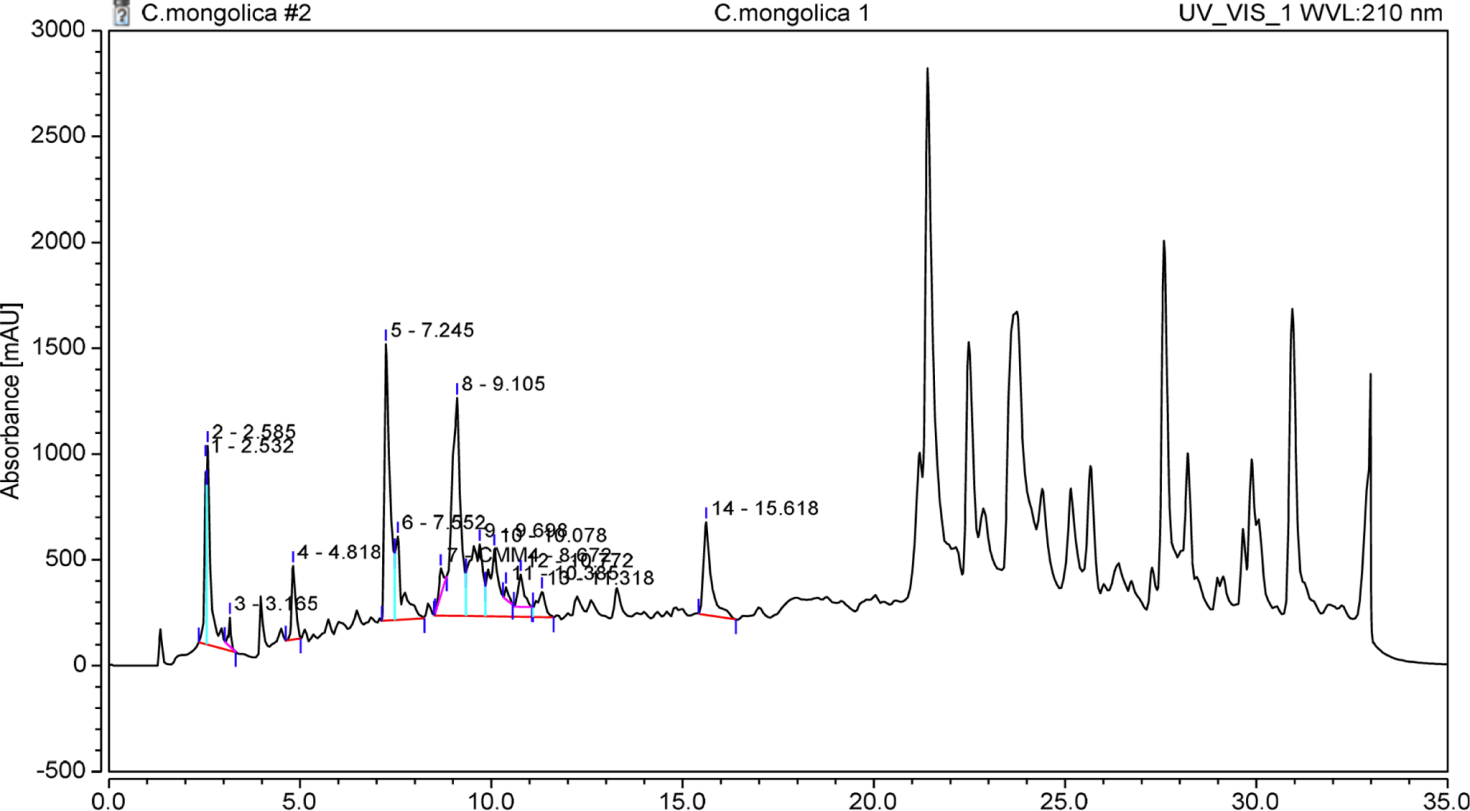
HPLC chromatogram of the root extract of *C. mongolica*, recorded at 210 nm.

To establish a chemical baseline for the plant material used in this study, an HPLC fingerprint of the *C. mongolica* root extract was recorded at 210 nm during a 35-minute chromatographic run (Fig. 2). The chromatogram revealed two broadly distinct, polarity-based regions: an early-eluting region (approximately 2.5−15.6 min) containing a series of well-defined, moderately intense peaks, including two prominent signals at 7.25 and 9.11 min, consistent with more hydrophilic constituents, followed by a more intense, later-eluting cluster (approximately 21.0−32.0 min) corresponding to the less polar, hydrophobic fraction of the extract. This elution pattern provides a reproducible chemical signature for the extract batch used in this study, reflecting a chemically complex matrix of constituents that may plausibly contribute to the antibacterial and wound-healing activities evaluated in the present study. Previous phytochemical investigations have indicated that the roots of this species are characterized by a rich matrix of specialized abietane diterpenoids, modified abeo-abietanes, and unique spiro-cyclopropane derivatives, offering a plausible chemical context for the polarity-based clustering observed in the present fingerprint.

### Determination of the Optimal Concentration

**Fig. 3:**
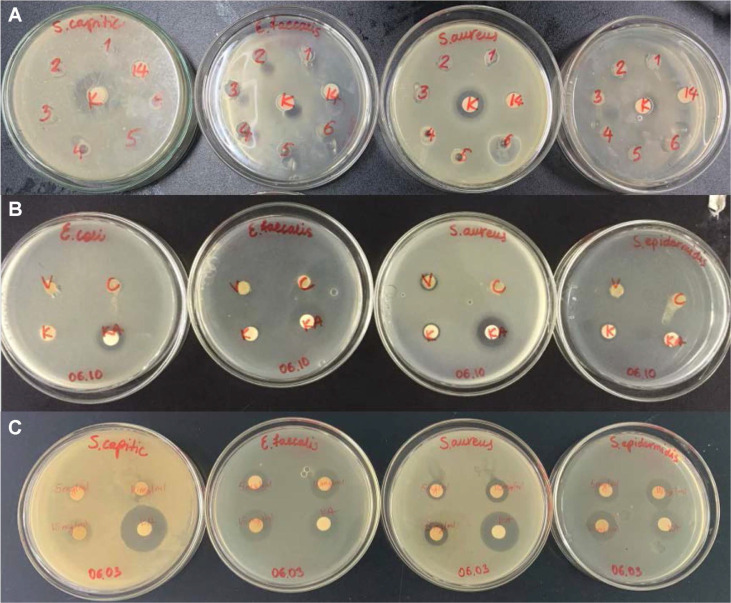
Antibacterial activity of the ointment formulations. (**A**) 1- Vaseline-based, 0.5 mg/mL *C. mongolica*; 2- Vaseline-based, 0.75 mg/mL *C. mongolica*; 3- Vaseline-based, 1.0 mg/mL *C. mongolica*; (**B**) V- Vaseline-based, 3.0 mg/mL *C. mongolica*; (**C**) Vaseline-based, 5.0 mg/mL *C. mongolica*; Vaseline-based, 10.0 mg/mL *C. mongolica*; and Vaseline-based, 15.0 mg/mL *C. mongolica*.

Vaseline-based formulations showed no detectable antibacterial activity at concentrations below 3.0 mg/mL. Inhibition zones were first observed at 3.0 mg/mL, indicating the onset of antibacterial activity. Consistent inhibition against all tested Gram-positive strains was achieved at 5.0 mg/mL (Table 1, Fig. 3). Although higher concentrations (10.0−15.0 mg/mL) produced larger inhibition zones for some strains, the increase was not proportional to the rise in extract concentration, indicating a diminishing dose-response relationship beyond 5.0 mg/mL. Given that 5.0 mg/mL already achieved consistent inhibition across all four tested strains, this concentration was selected as the minimum effective dose, balancing antibacterial efficacy, extract economy, and formulation practicality. Higher concentrations would substantially increase the amount of root extract required without a corresponding gain in antibacterial benefit. Therefore, 5.0 mg/mL was considered the optimal effective concentration for ointment formulation and was selected for further investigation.

### Physicochemical Evaluation of the *C. mongolica* Ointment

Physicochemical evaluation of the *C. mongolica* ointment demonstrated that all tested parameters were within the accepted limits specified for Vaseline in USP 30 standard (Table 2). The ointment appeared as a homogeneous, wax-like semi-solid substance, complying with the standard specification. It exhibited a mild characteristic odor consistent with the hydrocarbon-like odor specified in the USP 30 standard. The *C. mongolica* ointment was insoluble in both hot and cold water, confirming its hydrophobic nature. The melting point was determined to be 51°C, which falls within the specified range and the specific gravity measured at 100°C was 0.81 g/cm^3^, which also complied with the USP 30 specification.

### Microbiological results of *C. mongolica* Ointment

Microbiological evaluation of the *C. mongolica* ointment revealed that the total aerobic bacteria, yeast and mold, and Enterobacteriaceae counts were all measured at ≤10.0 CFU/g (detection limit), which is considerably lower than the permissible limits of ≤10^2^ CFU/g and ≤10^1^ CFU/g, respectively, as specified in Mongolian National Standards. Furthermore, neither *S. aureus* nor *P. aeruginosa* was detected in any of the tested samples. These results indicate that the formulation meets the microbiological quality requirements for non-sterile topical preparations and can be considered microbiologically safe for use (Table 3).

### Antibacterial Activity of *C. mongolica* Ointment

The antibacterial activity of the *C. mongolica* ointment was assessed by measuring the diameter of the inhibition zones, defined as clear areas of no visible bacterial growth surrounding the disks. The results demonstrated that the ointment retained antibacterial activity after both one week and one month of storage. Specifically, 5.0 mg/mL of the *C. mongolica* ointment retained moderate antibacterial activity against Gram-positive bacteria after one month of storage, with inhibition zone diameters comparable to those of the control sample containing 5.0 mg/mL of *C. mongolica* ethanol extract without the Vaseline base (Table 4).

### Wound Healing Potential

**Fig. 4:**
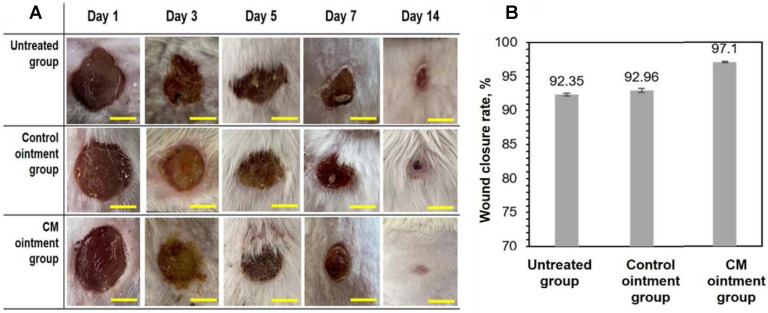
In vivo wound healing effects of *C. mongolica* ointment in mice. (**A**) Wound healing status of experimentally induced skin wounds in mice, and (**B**) Wound closure rate (%). Representative photographs were taken to document the *in vivo* wound closure process in a mouse model. A digital camera was utilized to capture images of untreated wounds (wounds that underwent surgery only), wounds treated with a control ointment, and wounds treated with *C. mongolica* ointment at 1, 3, 5, 7 and 14-days post-treatment. The scale bars in the photographs represent a measurement of 0.5 cm. Error bars represent SEM (n = 3 per group); the difference in wound closure rate among groups at day 14 did not reach statistical significance (one-way ANOVA, *p* = 0.728).

The wound healing efficacy of *C. mongolica* ointment was evaluated *in vivo* using a full-thickness excisional dorsal wound model in mice. Circular wounds were created on the dorsum of male BALB/c mice, which were randomly assigned to three groups: an untreated group, a group treated with a control ointment, and a group treated with the *C. mongolica* ointment. Representative macroscopic photographs documenting the progression of wound closure in each group are shown in Fig. 4, illustrating the overall morphological changes and wound contraction during the 14-day observation period.

Topical application of both ointments was associated with faster wound closure compared with the untreated group. In particular, mice treated with *C. mongolica* ointment demonstrated a visibly faster reduction in wound area and improved skin appearance, findings that were consistent with the histological evidence of re-epithelialization and tissue regeneration. On day 1, no significant differences were observed among the three groups. By day 3, both the control and *C. mongolica* ointment groups exhibited mild purulent discharge indicative of an early inflammatory response. On days 5 and 7, the control group displayed depressed wound morphology, while the untreated and control groups showed persistent yellowish pus accumulation, composed of dead leukocytes, bacterial debris, and serum- indicating ongoing infection and delayed wound repair.

By contrast, the *C. mongolica*-treated group exhibited visibly reduced inflammation and enhanced granulation tissue formation. By day 14, complete re-epithelialization was observed in this group, with smooth skin surface and no visible scar formation. Conversely, the untreated group retained slightly rough wound areas, and the control group showed depressed lesions, implying incomplete wound closure.

**Fig. 5:**
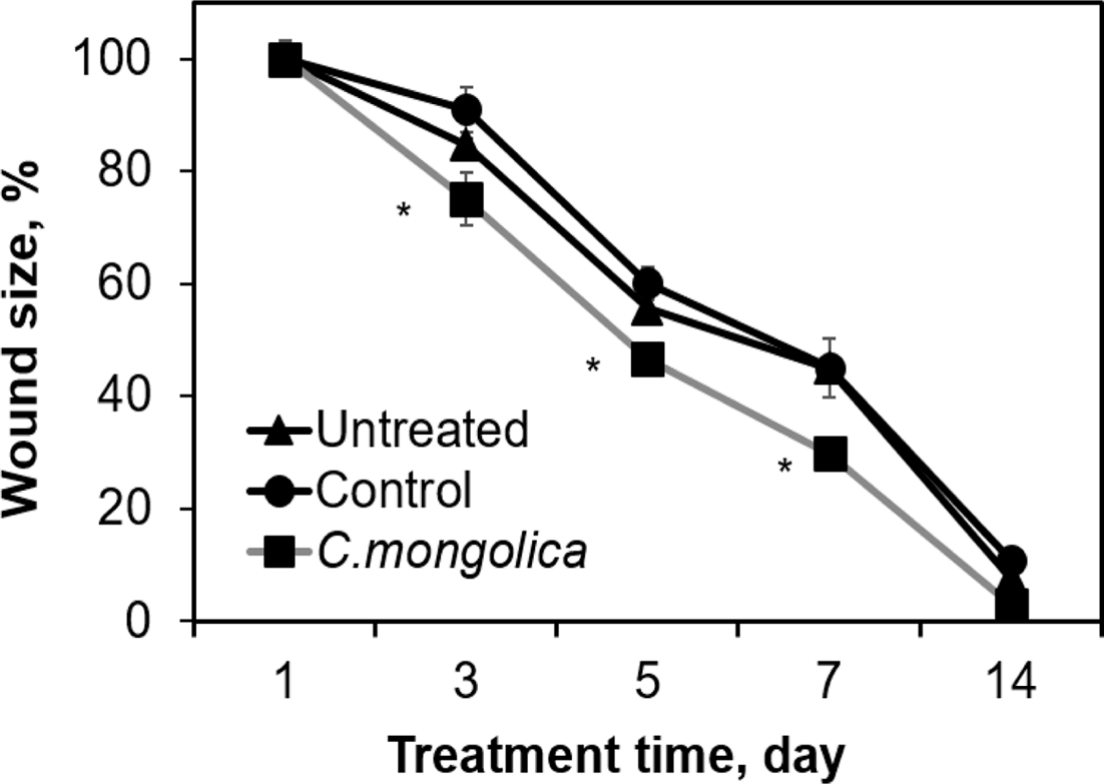
The wound healing potential was quantified by calculating the percentage of the wound area relative to its original size on day 14 after topical application. The wound area was measured using ImageJ. The untreated group ( ▲ ) consisted of mice that underwent surgery only, while the control group (●) received treatment with an ointment containing 5% ethanol, 1% lavender oil, and Vaseline. The *C. mongolica* group (■) received treatment with 5.0 mg/mL *C. mongolica* dissolved in the control ointment. The values are presented as mean ± SEM, with a sample size of 3 in each group. On days 3 and 5, the *C. mongolica*-treated group showed a statistically significant difference compared to the untreated and control groups (*p* = 0.023 and *p* = 0.016, respectively, one-way ANOVA with Tukey's post hoc test).

Quantitative wound area analysis was performed using ImageJ software at days 1, 3, 5, 7, 10, and 14. The results demonstrated that wound contraction occurred more rapidly in the *C. mongolica*-treated mice compared with the other groups. At the end of treatment, the mean wound closure rates were as follows: the untreated group achieved 92.35%, the control ointment group reached 92.96%, and the *C. mongolica*-treated group achieved the highest rate of 97.1% (Fig. 5).

By the end of the treatment, the body weight of the mice had increased by approximately 4% overall. When analyzed by group, the untreated group showed a weight increase of 3.8−4.9%, the control ointment group showed an increase of 3.2−5.3%, and the *C. mongolica*-treated group showed an increase of 4.1−7.8%. The absence of weight loss in the *C. mongolica*-treated group, comparable to that observed in both the untreated and control groups, indicates that this ointment did not exert any adverse effects on the animals.

Statistical analysis confirmed that the improvement observed in the *C. mongolica*-treated group was significant on days 3 and 5 (*p* = 0.023 and *p* = 0.016, respectively), consistent with more rapid early wound repair kinetics in this group. This difference did not reach statistical significance by day 7 (*p* = 0.185) or day 14 (*p* = 0.728), indicating that the gross wound area gap between groups narrowed as healing progressed in all groups.

### Histopathological Analysis

**Fig. 6:**
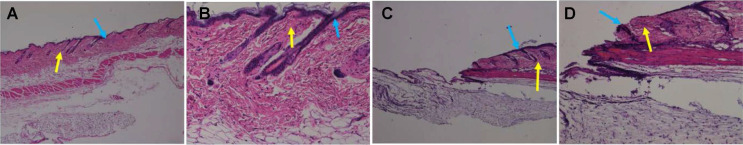
Histological features of wound healing in mouse dermal sections. The images (**A, B**) show healthy skin with intact epidermis and dermal layers, while images (**C, D**) show wounded skin with disrupted epidermis and dermal layers. The images were stained with hematoxylin and eosin. The blue arrow indicates the epidermis layer, and the yellow arrow points to a dermal layer of mouse skin. No scale bars are shown; images were acquired at 40× and 100× magnification.

**Fig. 7:**
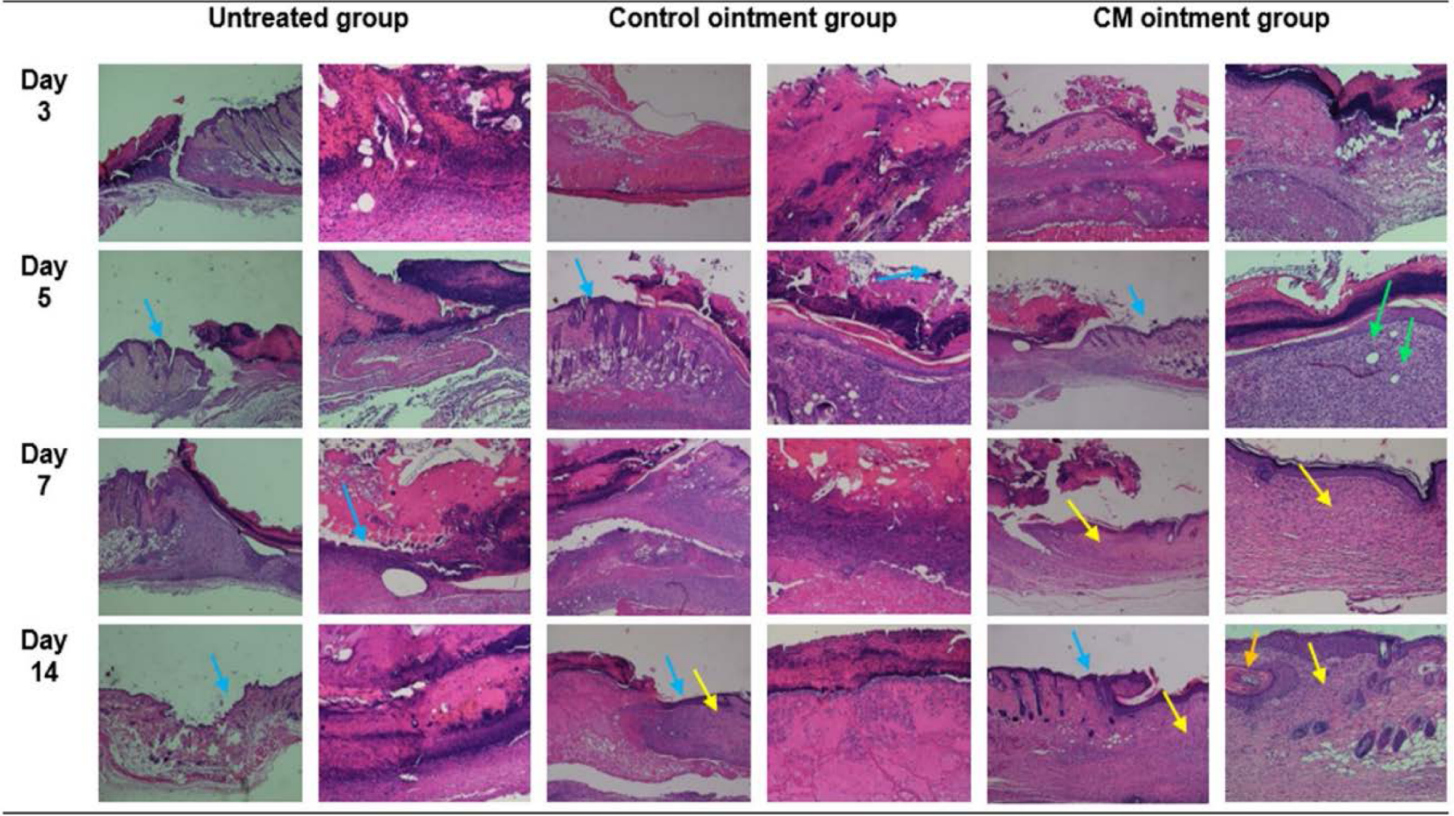
Histological features after treatment by using the effects of control ointment and *C. mongolica* ointment on excised wound tissues. Images were captured digitally using a microscope to track the growth rate of the new epithelial layer in each group. After 3, 5, 7, and 14 days of treatment, the untreated wound tissues were excised and sectioned. The images were stained with H&E at 40× and 100× magnification. The yellow arrow indicates a thin layer of collagen, the blue arrow represents keratinocytes, the orange arrow shows hair follicles, and the green arrow indicates neovascularization. No scale bars are shown; images were acquired at 40× and 100× magnification.

**Fig. 8:**
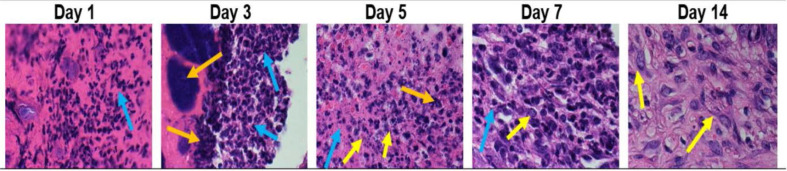
Histological features of neutrophils and mononuclear cells morphologically consistent with macrophages after 3, 5, 7, and 14 days of treatment with *C. mongolica* ointment. The samples were stained with H&E and viewed at 200× magnification. Neutrophil cells are indicated by the blue arrow, bacterial masses by the orange arrow, and mononuclear cells morphologically consistent with macrophages by the yellow arrow. No scale bars are shown; images were acquired at 200× magnification.

Macroscopic wound observations were supported by histopathological analyses conducted on skin tissues collected on days 1, 3, 5, 7, and 14 post-injury (Figs. 6−8). Quantitative metrics included the length of newly generated epidermis, granulation tissue thickness, semi-quantitative collagen scoring (0−4), vessel counts, and inflammatory-cell infiltration, providing a comprehensive evaluation of wound healing.

Microscopic evaluation of hematoxylin and eosin (H&E)-stained sections revealed distinct healing phases across the experimental timeline. On day 1, all groups exhibited superficial collagen at wound edges, damaged keratinocytes, edema, and fibrin deposition. Neutrophil infiltration was pronounced, indicating an acute inflammatory response. By day 3, edema and fibrin exudates began to resolve, particularly in the control ointment group, while inflammatory-cell infiltration remained elevated in all groups. In the *C. mongolica* ointment-treated group, neutrophil counts decreased and an increase in mononuclear cells morphologically consistent with macrophages was observed, a pattern indicative of a transition toward tissue remodeling.

On day 5, collagen fibers in the control groups remained irregular (score 1-2), whereas treated wounds displayed moderately organized collagen (score 2−3). Early neovascularization was observed in the treated group, consistent with the initiation of vascular remodeling. On day 7, keratinocyte layers were re-established in treated wounds, accompanied by reduced edema and compact dermal collagen. Granulation tissue thickness was greater in treated wounds compared with controls. Necrotic foci and persistent mononuclear infiltration were still observed in untreated wounds (Fig. 7).

On day 14, quantitative histological analysis revealed marked differences between the *C. mongolica*-treated and control groups, with the corresponding histological parameters summarized in Table 5. *C. mongolica*-treated wounds demonstrated significantly greater epidermal length (794.65 ± 75.27 μm vs. 394.59 ± 58.5 μm), granulation tissue thickness (558.02 ± 65.79 μm vs. 349.36 ± 23.69 μm), collagen score (3−4 vs. 1−2), and vessel counts (16.2 ± 4.99 vs. 3.75 ± 1.6 per HPF) than control wounds, while inflammatory cell infiltration was markedly reduced (score 1 vs. 3; all *p* < 0.05) (Fig. 8).

Overall, these findings quantitatively and qualitatively indicate that *C. mongolica* ointment treatment was associated with enhanced epithelial regeneration, granulation tissue formation, collagen maturation, neovascularization, and more favorable resolution of inflammation compared with controls.

## Discussion

This study provides evidence for the wound-healing potential of *C. mongolica* extract, consistent with its traditional use in Mongolian, Tibetan, and Chinese medicine for bacterial infections and its previously reported anti-Brucella activity [21, 22, 29]. The bioactive compound classes identified in this species, including alkaloids, flavonoids, diterpenoid glycosides, and iridoid glucosides [19, 23, 26, 29], are consistent with the wound-healing outcomes observed in the present study; however, whether these effects operate through mitigation of microbial proliferation was not directly assessed. Consistent with these reports, the HPLC fingerprint obtained for the extract batch used in the present study (Fig. 2) confirmed a chemically complex profile spanning both hydrophilic and hydrophobic compound classes. This phytochemical profile is consistent with the antibacterial activity and wound-healing outcomes observed in the present study and provides a reproducible reference signature for future investigations using this extract. Notably, while the crude ethanolic extract demonstrated inhibitory activity against *E. faecalis*, the ointment formulation did not produce a measurable inhibition zone against this organism (Table 4). This discrepancy may be attributed to the hydrophobic nature of the Vaseline base, which can limit the diffusion of bioactive compounds into the agar medium, thereby reducing the apparent activity in disk diffusion assays. Similar matrix-dependent reductions in antibacterial efficacy have been reported for other petrolatum-based topical formulations. Our study has several limitations. The control ointment contained lavender oil, which itself has antimicrobial and wound-healing activity [33]; therefore, the *C. mongolica*-specific effect should be interpreted conservatively. The wound model was not experimentally infected, so the antibacterial contribution to healing is inferred rather than directly measured. Antibacterial activity was assessed only by disk diffusion rather than MIC/MBC. Future studies incorporating a Vaseline-only control, an infected wound model, and MIC/MBC determination would address these limitations. Indeed, natural products with anti-inflammatory, antioxidant, and antibacterial activities are well documented to be associated with accelerated wound healing, through mechanisms that may include modulation of inflammatory responses, promotion of fibroblast proliferation, stimulation of collagen deposition, and enhancement of epithelialization [3-8, 34]. Wound healing is a highly coordinated, dynamic process comprising sequential yet overlapping phases, including hemostasis, inflammation, proliferation, fibroplasia, neovascularization, granulation tissue formation, re-epithelialization, and remodeling of the extracellular matrix [5, 35].

In our study, macroscopic and histological observations revealed that wounds treated with *C. mongolica* ointment exhibited accelerated closure and improved tissue architecture compared to the control ointment and untreated groups. On day 3, both ointment-treated groups displayed inflammatory responses and edema, as expected during the early phase of wound healing. By day 14, the *C. mongolica*-treated wounds demonstrated nearly complete re-epithelialization, organized dermal architecture, abundant collagen deposition, and well-developed adnexal structures, with minimal inflammatory cell infiltration. In contrast, the control and untreated groups exhibited delayed healing, persistent edema, and incomplete epidermal regeneration. These findings are consistent with the possibility that the antibacterial activity of *C. mongolica* may have contributed to a more favorable healing trajectory observed in this group; however, this relationship was not directly demonstrated, as bacterial burden was not quantified in the wound tissue (Figs. 4−5).

Histopathological analysis further confirmed these observations. The untreated group showed delayed epidermal regeneration, persistent inflammatory infiltrates, and less organized dermal collagen deposition throughout the observation period. In the control ointment group, inflammation, edema, and fibrin deposits were initially prominent but decreased over time, with moderate collagen deposition and neovascularization evident by day 14. In contrast, the *C. mongolica* ointment group exhibited early keratinocyte regeneration, enhanced neovascularization, histological features consistent with increased fibroblast activity, and well-organized collagen fibers as early as day 7, with minimal neutrophil and mononuclear cell infiltration by day 14 (Figs. 6−8). These histological findings are consistent with more favorable inflammation resolution, neovascularization, collagen synthesis, and tissue remodeling in the *C. mongolica*-treated group relative to controls. As these observations are based on H&E staining and semi-quantitative scoring rather than molecular markers, they should be interpreted as descriptive histological evidence rather than direct confirmation of underlying molecular mechanisms.

Collagen deposition and maturation are critical determinants of wound tensile strength and functional recovery [36, 37]. In the *C. mongolica*-treated group, wounds showed improved granulation tissue formation, consistent with increased collagen deposition observed histologically. The greater granulation tissue thickness and increased collagen content observed in *C. mongolica*-treated wounds are consistent with enhanced mechanical stability of the repaired tissue. Such observations align with previous reports on the fibroblast-stimulating and extracellular matrix-stabilizing properties of natural phytochemicals [38, 39]. Additionally, macrophages play a central role in orchestrating wound healing by producing growth factors, cytokines, and chemokines that regulate inflammation, phagocytosis, angiogenesis, and tissue remodeling. In the *C. mongolica*-treated group, the temporal decrease in neutrophils and concomitant increase in mononuclear cells morphologically resembling macrophages are consistent with a possible transition from the inflammatory to the proliferative phase. However, macrophage identity was not confirmed by specific immunohistochemical markers.

Quantitative histological analysis at day 14 confirmed that the treated group achieved nearly complete re-epithelialization (794.65 ± 75.27 μm) compared with incomplete closure in controls (394.59 ± 58.5 μm, *p* < 0.05). Granulation tissue thickness, collagen deposition, and vessel counts were significantly higher in treated wounds, indicating enhanced dermal matrix maturation and neovascularization. Additionally, inflammatory-cell infiltration was minimal in treated wounds (score 1), but persisted in controls (score 3), suggesting more favorable resolution of inflammation in the treated group. Together, these findings suggest that *C. mongolica* ointment is associated with favorable progression through the wound-healing cascade, from inflammation to proliferation to remodeling. Collectively, our findings indicate that topical application of *C. mongolica* extract was associated with improved wound-healing outcomes, accompanied by histological features consistent with antibacterial, anti-inflammatory, and pro-regenerative activity. This study provides histological and macroscopic evidence that *C. mongolica* ointment was associated with faster wound closure and favorable epithelial regeneration, neovascularization, and dermal remodeling, supporting its potential as a natural wound-healing candidate. Further mechanistic studies incorporating molecular markers (*e.g.*, VEGF, CD31, collagen I/III, TNF-α, IL-6) are warranted to confirm the specific molecular pathways by which *C. mongolica* phytochemicals regulate cellular responses during dermal repair.

In our study, a *C. mongolica* root extract-based ointment was developed through systematic formulation optimization and evaluated within an integrated antibacterial-wound healing framework using a murine excisional wound model. Topical application of the formulation was associated with faster wound closure, greater re-epithelialization, increased collagen deposition, and more pronounced neovascularization compared with untreated and control groups. Histopathological analyses revealed reduced neutrophil infiltration, an increase in mononuclear cells morphologically consistent with macrophages on H&E staining, and well-organized granulation tissue in the treated group. Taken together, the data support an association between *C. mongolica* ointment treatment and improved wound-healing outcomes; however, a direct mechanistic link between the antibacterial activity of the extract and wound repair was not demonstrated in this study. These findings are consistent with both antibacterial activity and wound-healing potential of a standardized *C. mongolica* root extract ointment in an experimental animal model. The results are in line with its traditional use and suggest its potential as a natural formulation candidate for wound management. It should be noted that the vehicle control ointment contained lavender essential oil, which itself possesses antimicrobial and wound-healing properties. Therefore, the specific biological contribution of the *C. mongolica* extract cannot be fully distinguished from that of the vehicle components, and the observed wound-healing effects should be interpreted with this limitation in mind. Future studies should focus on elucidating the underlying molecular mechanisms, evaluating extract standardization and batch-to-batch reproducibility, assessing dermal toxicity and biocompatibility, and validating efficacy in an infected wound model. Such investigations would constitute a necessary foundation before any consideration of clinical translation.

## Figures and Tables

**Table 1:** Antibacterial activity results of *C. mongolica* Vaseline-based formulations.

Sample name and concentration	Zone of inhibition (mm)			
	*E. faecalis*	*S. aureus*	*S. epidermidis*	*M. luteus*
Vaseline-based, 0.5 mg/mL	-	-	-	-
Vaseline-based, 0.75 mg/mL	-	-	-	-
Vaseline-based, 1.0 mg/mL	-	-	-	-
Vaseline-based, 3.0 mg/mL	11	10.5	12.5	14
Vaseline-based, 5.0 mg/mL	14	15	12	16
Vaseline-based, 10.0 mg/mL	16	12	16	15
Vaseline-based, 15.0 mg/mL	17	13	17	17
Kanamycin, 0.5 mg/mL	20	18	11	12.5

Note: Values represent zones of inhibition in millimeters (mm). (-) indicates no activity detected.

**Table 2:** Physicochemical parameters of *C. mongolica* ointment.

Parameter	Standard specification	Test result
Appearance	Colorless, liquid or wax-like substance	Complies
Odor	Hydrocarbon-like odor	Characteristic odor
Solubility	Insoluble in hot and cold water	Insoluble in water
Melting point (°C)	38−60	Complies
Specific gravity, g/cm^3^ (100°C)	0.75−0.87	Complies

**Table 3:** Microbiological test results of *C. mongolica* ointment.

Tests	Specification	Results
The total aerobic bacteria count	NMT 10^2^ CFU/g	NMT 10.0
Yeast and Molds counts	≤10^2^ CFU/g	≤10 CFU/g
Enterobacteriaceae counts	NMT 10^1^ CFU/g	NMT 10.0
*Staphylococcus aureus*	Not detected	Not detected
*Pseudomonas aeruginosa*	Not detected	Not detected

Note: NMT- Not More Than, CFU/g - Colony Forming Units per gram

**Table 4:** Antibacterial activity results of *C. mongolica* ointment.

No.	Sample name	Zone of inhibition (mm)			
		*E. faecalis*	*S. aureus*	*S. epidermidis*	*M. luteus*
1	*C. mongolica* ethanol extract	10	10	11	10
2	5.0 mg/mL *C. mongolica* ointment	-	9.5	9.5	10
3	5.0 mg/mL *C. mongolica* ointment, after 7 days	-	10	8	8.5
4	5.0 mg/mL *C. mongolica* ointment, after 1 month	-	10	9	10

Note: Values represent zones of inhibition in millimeters (mm). (-) indicates no activity detected.

**Table 5:** Quantitative histological parameters of wound healing on day 14.

Parameter	Control ointment	*C. mongolica* ointment	*p*-value
Epidermal length (μm)	394.59 ± 58.5	794.65 ± 75.27	< 0.05
Granulation tissue thickness (μm)	349.36 ± 23.69	558.02 ± 65.79	< 0.05
Collagen score (0−4)	1−2	3−4	< 0.05
Vessel count (per HPF)	3.75 ± 1.6	16.2 ± 4.99	< 0.05
Inflammatory cell score (0−4)	3	1	< 0.05

Note: HPF: high-power field. Collagen and inflammatory cell infiltration were assessed using a semi-quantitative scoring system (0−4). Values are expressed as mean ± SD.
